# Supplementary material for: Differences in water and vapor transport through angstrom-scale pores in atomically thin membranes
Source: Nat Commun. 2022 Nov 7;13:6709. doi: 10.1038/s41467-022-34172-1 (PMC9640652; doi:10.1038/s41467-022-34172-1)
Supplement: Supplementary file 1 — Supplementary Information [file 41467_2022_34172_MOESM1_ESM.pdf]

Supplementary Information

Differences in Water and Vapor Transport through Angstrom-scale Pores in Atomically Thin Membranes

Peifu Cheng,[1] Francesco Fornasiero,[2] Melinda L. Jue,[2] Wonhee Ko,[3] An-Ping Li,[3] Juan Carlos Idrobo,[3,7] Michael S. H. Boutilier,[4] and Piran R. Kidambi[1,5,6*]

[1]*Department of Chemical and Biomolecular Engineering, Vanderbilt University, Nashville, Tennessee 37212, United States.*

[2]*Physical and Life Sciences, Lawrence Livermore National Laboratory, Livermore, California 94550, United States.*

[3]*Center for Nanophase Materials Sciences, Oak Ridge National Laboratory, Oak Ridge, Tennessee 37831, United States.*

[4]*Department of Chemical and Biochemical Engineering, Western University, London, Ontario N6A 5B9, Canada.*

[5]*Department of Mechanical Engineering, Vanderbilt University, Nashville, Tennessee 37212, United States.*

[6]*Vanderbilt Institute of Nanoscale Sciences and Engineering, Vanderbilt University, Nashville, Tennessee 37212, United States.*

[7]*Materials Science and Engineering Department, University of Washington, Seattle, WA 98195, USA*

[*]Email piran.kidambi@vanderbilt.edu

This Supplementary Information includes:

- Supplementary Figures 1 to 17
- Supplementary Tables 1 to 3
- Supplementary Notes 1 to 2
- Supplementary References

Supplementary Table 1 Water transport rates reported in the literature.

Membrane	Reported water transport rate	Converted water permeance		Pore size (nm)	Reference
		$L\ m^{-2}\ h^{-1}\ bar^{-1}$	$g\ m^{-2}\ day^{-1}\ Pa^{-1}$		
Graphene	10-130 $L\ cm^{-2}\ day^{-1}\ MPa^{-1}$	417-5417	100-1300	0.15-0.89	15
TFC-RO	1-12 $L\ m^{-2}\ h^{-1}\ bar^{-1}$	1-12	0.24-2.88	~0.5 nm	7
Graphene	$2.7\times10^{-8}\ m^3\ m^{-2}\ s^{-1}\ Pa^{-1}$	9720	~2300	50 nm	40
Graphene*	$10^6\ g\ m^{-2}\ s^{-1}$ at 17 kPa	$\sim3\times10^{10}$	5×10^6	<1 nm	30
Graphene**	$10^6\ g\ m^{-2}\ s^{-1}$ at 17 kPa	$\sim2.1\times10^7$	5×10^6	<1 nm	30
Graphene	70 $g\ m^{-2}\ s^{-1}\ atm^{-1}$	~250	~60	<1 nm	30
GNM/SWNT	20.6 $L\ m^{-2}\ h^{-1}\ bar^{-1}$	20.6	~5	<1.2 nm	31
GNM/SWNT	97.6 $L\ m^{-2}\ h^{-1}\ bar^{-1}$	97.6	~23	<1.2 nm	31
CNM*	1.1×10^{-4} mol $m^{-2}\ s^{-1}\ Pa^{-1}$	9.85×10^5	~170	<1.1 nm	18
CNM**	1.1×10^{-4} mol $m^{-2}\ s^{-1}\ Pa^{-1}$	713	~170	<1.1 nm	18

* Assuming water vapor tranport through the membrane.
** Assuming liquid water tranport through the membrane.

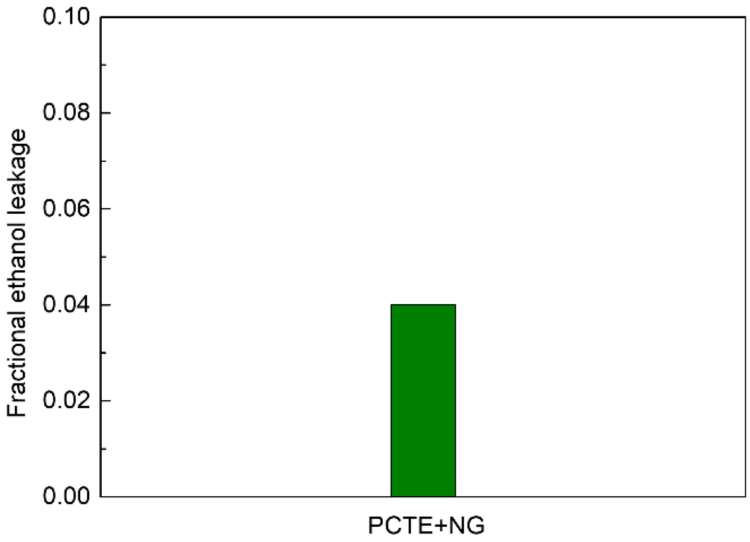

Supplementary Figure 1. Fractional leakage (PCTE+NG / (PCTE)) of ethanol across nanoporous graphene (NG) on PCTE support (PCTE+NG). The ~4% of fractional ethanol leakage indicates ~96% graphene coverage.

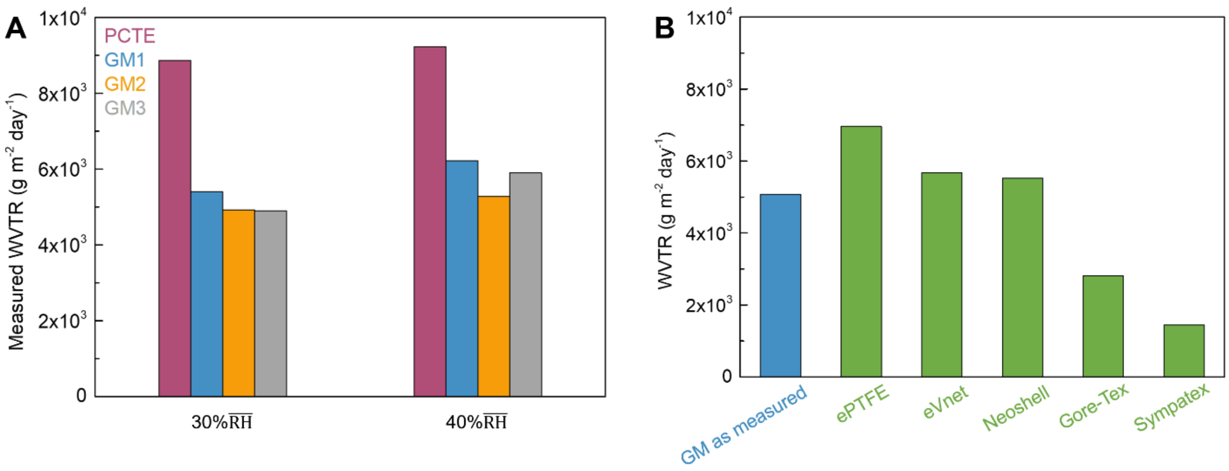

Supplementary Figure 2. (A) Measured WVTRs through PCTE and the fabricated GMs under different mean relative humidity (30% and 40%). The measured WVTR is calculated without accounting for ~9.4% porosity of PCTE supports. (B) WVTRs of the fabricated GM without accounting for ~9.4% porosity of PCTE supports and some commercial breathable materials[1] (ePTFE, eVent, Neoshell, Gore-Tex, and Sympatex) measured under 30% $\overline{RH}$ with a constant RH gradient of 50% across the membrane at 30 °C.

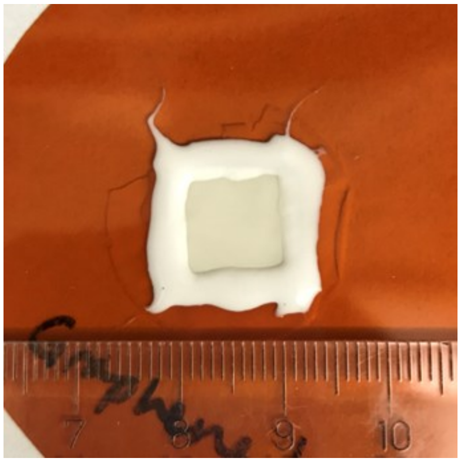

Supplementary Figure 3. Optical image of a fabricated graphene membrane (mounted to an orange polyimide frame with white epoxy) subjected to water vapor transport measurements. The area for measurement is a large square with each side ~0.9 cm.

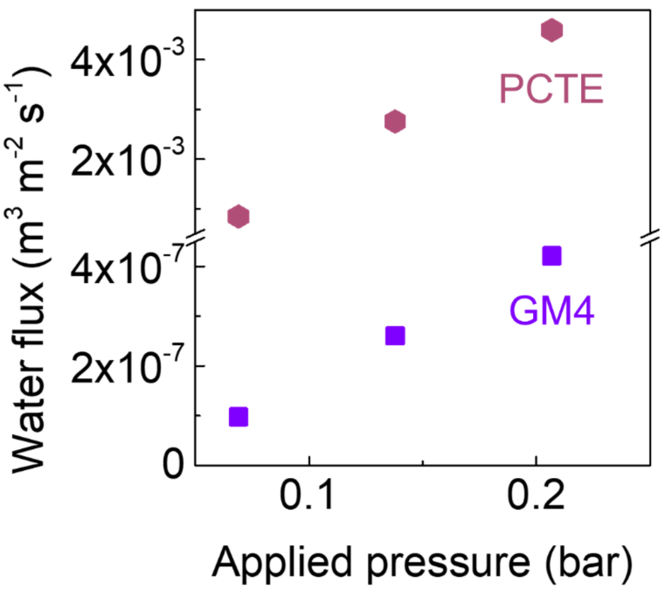

Supplementary Figure 4. Water flux measured through bare PCTE and graphene membrane (GM) under reverse osmosis (RO) with unit conversion from Fig. 2H to facilitate comparison with Fig. 2E.

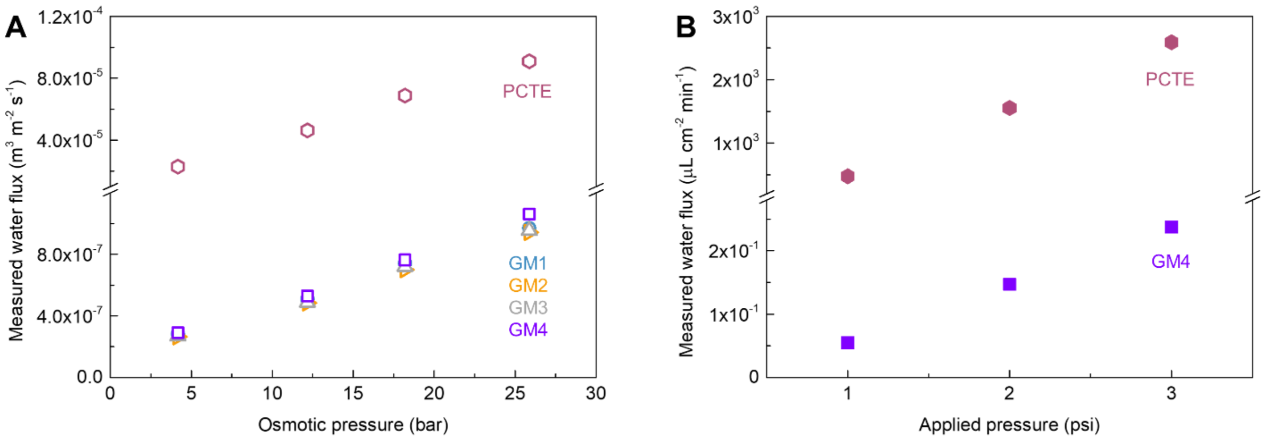

Supplementary Figure 5. A) Measured water flux across PCTE and the fabricated GMs under forward osmosis. B) Measured water flux across PCTE and the fabricated GM under reverse osmosis. Note water fluxes are calculated without accounting for ~9.4% porosity of PCTE supports.

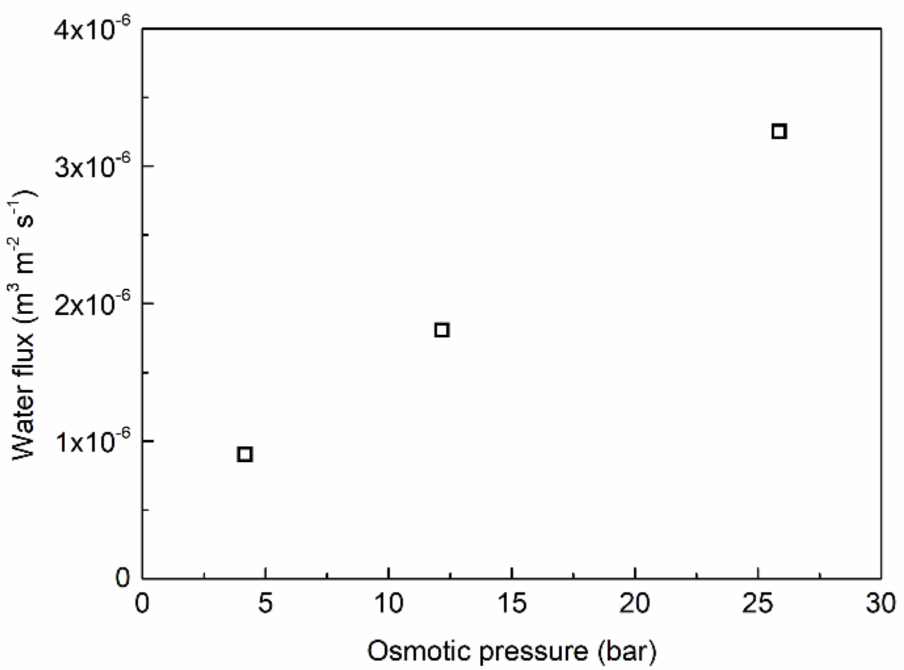

Supplementary Figure 6. Water flux calculated for PCTE+IP membrane (~0.19 g m^{-2} day^{-1} Pa^{-1} accounting for ~9.4% porosity of PCTE supports) under forward osmosis (FO).

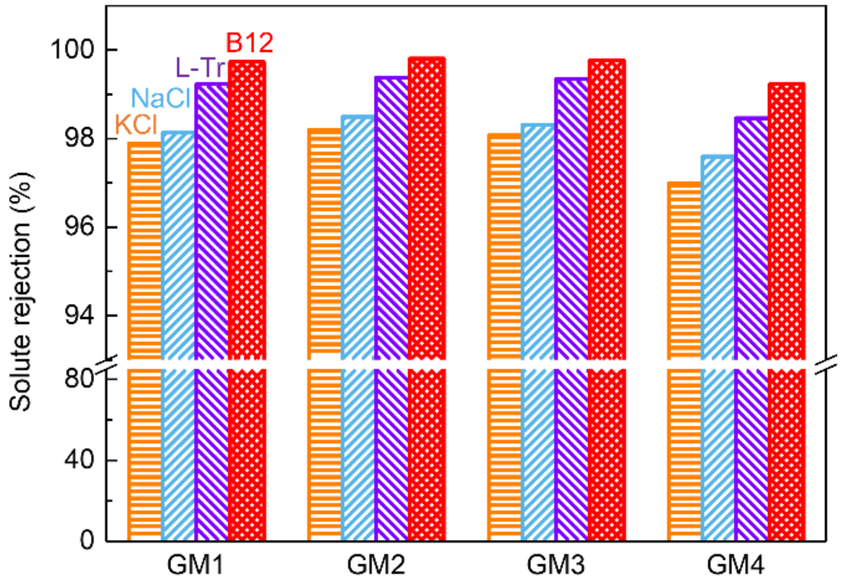

Supplementary Figure 7. Solute rejections of KCl, NaCl, L-Tr, and B12 through the fabricated GMs. The solute rejection was also calculated by another equation,[2] $S_{rejection} = \left(1 - \frac{C_p}{C_f}\right) \times 100\%$, where C_p is the solute concentration on permeate side after 24 h, and C_f is the initial solute concentration on feed side.

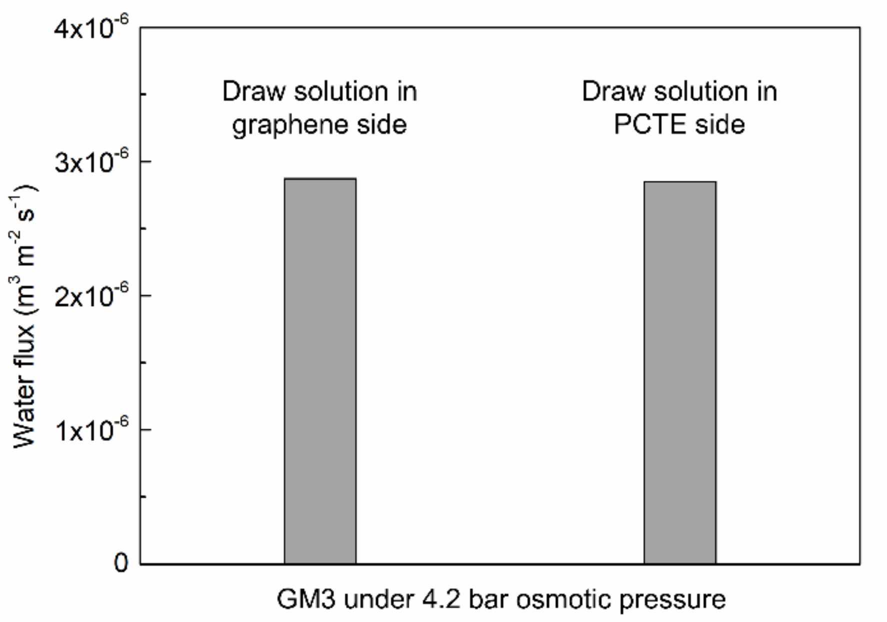

Supplementary Figure 8. Water flux across angstrom scale pores in graphene membrane (GM3) when draw solution (glycerol ethoxylate, molecular diameter ~1.2 nm) is filled in the graphene side versus the PCTE side.

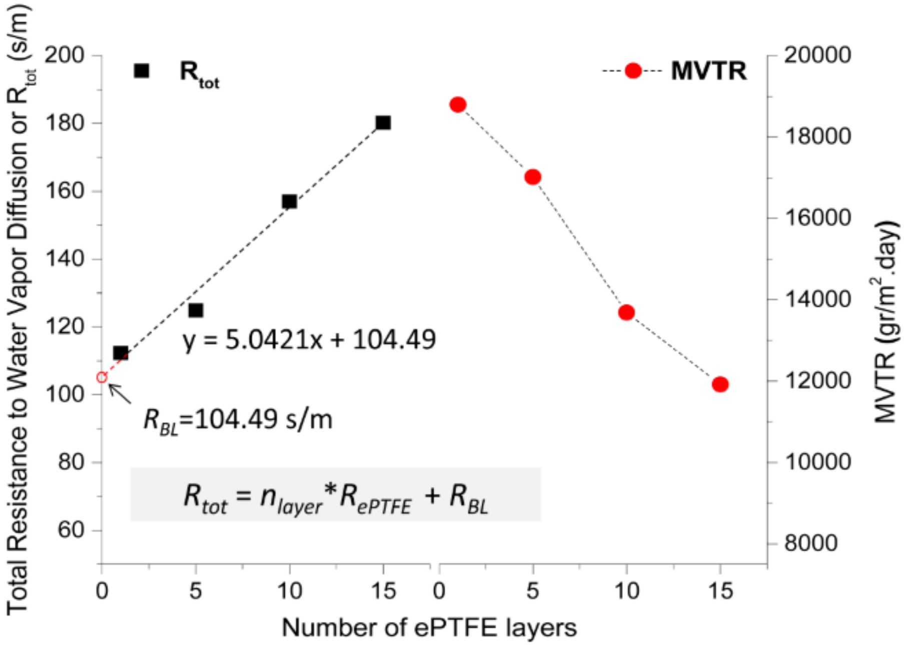

Supplementary Figure 9. Boundary layer resistance (~104.5 s/m) of the DMPC system during water vapor transport measurements. Reproduced with the permission from Ref. 1, Copyright 2016, John Wiley & Sons.

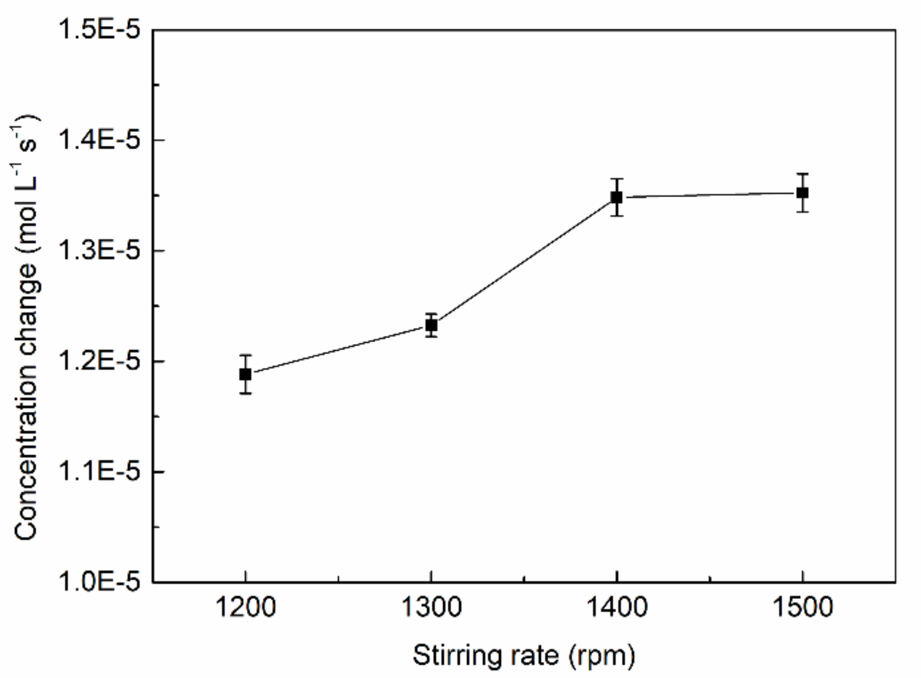

Supplementary Figure 10. KCl concentration in the permeate side of the diffusion system as a function of stirring rate. The plateau beyond 1400 rpm indicates minimization of concentration polarization effects.

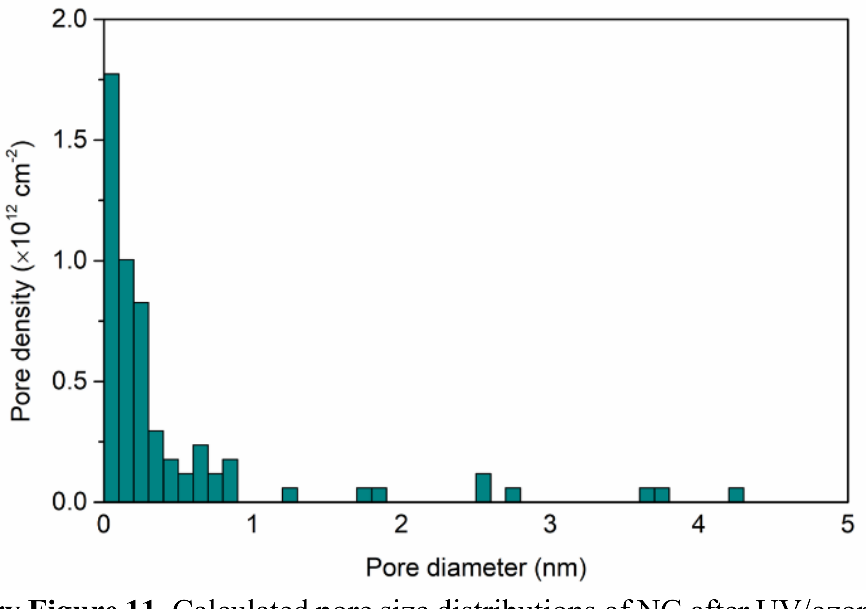

Supplementary Figure 11. Calculated pore size distributions of NG after UV/ozone treatment for 25 min.[3–6] The calculated pore diameter was obtained by adding carbon electron diameter (~0.13 nm) to the measured pore diameter, and then subtracting carbon van der Waals diameter (~0.34 nm). The comparison between measured (Fig. 1F) and calculated pore size distributions (Supplementary Figure 11) is consistent with the prior reports.[3–6]

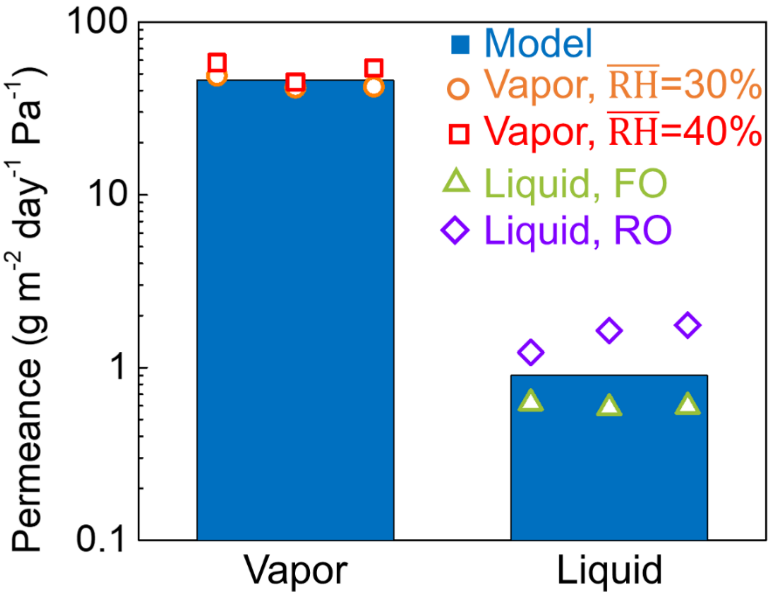

Supplementary Figure 12. Comparison of the transport model with the measured liquid and vapor permeances shown in Fig. 3F with a logarithmic Y-axis.

Supplementary Note

Supplementary Note 1. Analytical transport models

This section provides a detailed explanation of the transport models discussed in the main paper.

Supplementary Note 1.1 Pore size distribution

The pore size distribution in graphene was measured by STEM imaging of 89 pores. The observed open area of the pore was measured from these images and the diameter of a circle with that same area calculated. The apparent carbon diameter in STEM images depends on the accelerating voltage and does not reflect the van der Waals diameter of the carbon atoms relevant to transport. To correct for this, the apparent carbon diameter observed in STEM (0.13 nm) was added to the calculated pore diameter, and the van der Waals diameter of carbon (0.34 nm) was subtracted.[3] The size of the passing water molecule also reduces the effective pore diameter available for transport. To compensate for this, the mean van der Waals diameter of water (0.28 nm) was also subtracted from the pore diameter.[3] This gives an estimate of the size distribution of pores permeable to water. When adjusted in this way, pores with negative diameters are not permeable to water and are excluded from the distribution. Furthermore, POSS molecules seal larger pores. Although there is uncertainty in the smallest pore diameter that POSS will plug, here we exclude pores larger than 0.8 nm to match measured flow rates.

With these adjustments, the measured pore density is $n = 0.95 \times 10^{12}$ pores/cm^2. The average effective pore diameter is,

$$\langle D \rangle = \frac{\sum D}{N_{pore}} = 0.23 \text{ nm} \tag{S1}$$

where D is the diameter of one specific nanopore, N_{pore} is the number of water permeable pores, and the summation here and throughout is over all water permeable pores. Similarly,

$$\langle D^2 \rangle = \frac{\sum D^2}{N_{pore}} = 0.071\ \mathrm{nm}^2 \tag{S2}$$

$$\langle D^3 \rangle = \frac{\sum D^3}{N_{pore}} = 0.026\ \mathrm{nm}^3 \tag{S3}$$

Supplementary Note 1.2 Vapor transport model

Vapor transport is modeled as free molecular flow in both the graphene pores and the PCTE membrane pores. However, we note that the presence of nitrogen in the PCTE membrane pores may reduce the mean free path of water vapor enough that the Knudsen number is not quite in the ballistic regime. We accepted the potential error incurred by this approximation to arrive at a simpler expression for the flow rate and because it still captures the physical phenomena responsible for the significant difference in vapor and liquid phase flow rates.

Vapor flow through the support PCTE membrane pore is modeled as Knudsen diffusion with a mass flow rate of,[7]

$$\dot{m}_{supp} = \frac{3}{8}\frac{D_{supp}\Delta P\ A_{supp}}{L_{supp}}\sqrt{\frac{m}{2\pi k_B T}} \tag{S5}$$

where ΔP is the water vapor partial pressure difference across the pore, m is the mass of a water molecule, $L_{supp} = 10$ µm is the length of the support PCTE pore (Fig. 3D), $D_{supp} = 0.2$ µm is the PCTE membrane pore diameter, $A_{supp} = \pi D_{supp}^2/4$ is the cross sectional area of a PCTE membrane support pore, k_B is Boltzmann's constant, and T is temperature.

Flow through each graphene pore is modeled as Knudsen effusion with mass flow rate,[8]

$$\dot{m}_{pore} = \Delta P \frac{\pi}{4} D^2 \sqrt{\frac{m}{2\pi k_B T}} \tag{S6}$$

where D is the diameter of the graphene pore. The size of graphene pores varies significantly. Furthermore, each PCTE membrane pore has many pores in the graphene over it. The total flow rate through the graphene over a PCTE membrane pore is the sum of the flow rates through each of the graphene pores. We account for this by computing an average total flow rate through the graphene over a single PCTE membrane pore by summing the mass flow rate through a single graphene pore over the pore size distribution:

$$\dot{m}_{NG} = nA_{supp}\frac{\sum \dot{m}_{pore}}{N_{pore}} \tag{S7}$$

where n is the average number of water permeable graphene pores per unit area. Substituting in the expression for $\dot{m}_{pore}$,

$$\dot{m}_{NG} = nA_{supp}\Delta P\frac{\pi}{4}\langle D^2\rangle\sqrt{\frac{m}{2\pi k_B T}} \tag{S8}$$

By conservation of mass for the steady flow through this membrane, the flow rate through the PCTE pore must be the same as that through the graphene pores over it. Part of the pressure drop between the upstream and downstream side of the membrane occurs in the PCTE pore while the rest occurs across the graphene. We calculate the total flow rate using an equivalent flow rate resistance model, where current is replaced by mass flow rate and the voltage difference is replaced by the pressure difference. The mass flow rate expressions all have the form,

$$\dot{m} = \frac{\Delta P}{R} \tag{S9}$$

where R is the flow rate resistance. From Equation S5, the PCTE membrane pore resistance is,

$$R_{supp} = \frac{8}{3}\frac{L_{supp}}{A_{supp}D_{supp}}\sqrt{\frac{2\pi k_B T}{m}} \tag{S10}$$

Similarly, from Equation S8, the graphene resistance is,

$$R_{NG} = \frac{4}{\pi}\frac{1}{nA_{supp}\langle D^2\rangle}\sqrt{\frac{2\pi k_B T}{m}} \tag{S11}$$

The total flow rate through the membrane is found by adding the PCTE support pore resistance and graphene pore resistance in series,

$$\dot{m}_{NG+supp} = \frac{\Delta P}{R_{supp} + R_{NG}} \tag{S12}$$

Substituting in Equation S10 and S11 leads to a permeance of,

$$\frac{\dot{m}_{NG+supp}}{\Delta P\, A_{supp}} = \frac{\sqrt{\frac{m}{2\pi k_B T}}}{\frac{8}{3}\frac{L_{supp}}{D_{supp}} + \frac{4}{\pi}\frac{1}{n\langle D^2\rangle}} \tag{S13}$$

Plugging in values, we obtain a water vapor permeance of,

$$\frac{\dot{m}_{NG+supp}}{\Delta P\, A_{supp}} = 46\ \frac{\mathrm{g}}{\mathrm{m^2\ day\ Pa}} \tag{S14}$$

In reasonable agreement with our measurements.

Supplementary Note 1.3 Liquid transport model

Liquid flow through the PCTE support pores is modeled as continuum Poiseuille flow, *i.e.*, fully developed laminar pipe flow. In this case the mass flow rate through a support pore is,[9]

$$\dot{m}_{supp} = \frac{\Delta P \rho D_{supp}^2 A_{supp}}{32\mu L_{supp}} \tag{S15}$$

where $\rho = 1000$ kg/m^3 and $\mu = 0.001$ Pa-s are the density and viscosity of water, respectively. The equivalent hydrodynamic resistance is thus,

$$R_{supp} = \frac{32\mu L_{supp}}{\rho D_{supp}^2 A_{supp}} \tag{S16}$$

Flow through each graphene pore is also modeled as continuum flow using Sampson's equation for pressure driven creeping flow through an infinitesimal thickness orifice plate,[10]

$$\dot{m}_{pore} = \frac{\Delta P D^3 \rho}{24\mu} \tag{S17}$$

We note that Suk & Aluru[11] developed a correlation for water flux through graphene nanopores by fitting molecular dynamics simulation results. Their correlation accounts for deviations from continuum behavior for small pores. However, the differences produced by these effects are small compared to the flow rate changes when comparing vapor and liquid transport. We have thus decided to use the simpler expression in Equation S17 because it captures the important differences in transport with a less complicated expression. The total flow rate through the graphene area over a single support pore is calculated by substituting Equation S17 into Equation S7,

$$\dot{m}_{NG} = nA_{supp}\frac{\Delta P \langle D^3\rangle \rho}{24\mu} \tag{S18}$$

The equivalent resistance is thus,

$$R_{NG} = \frac{24\mu}{n\rho A_{supp}\langle D^3\rangle} \tag{S19}$$

The graphene and support pore also act in series for liquid flow. The resistance model is still that in Fig. 3E with the combined resistance given by Equation S12, but with the resistance for the graphene and support replaced by the liquid flow resistances in Equation S16 and S19. Thus,

$$\frac{\dot{m}_{NG+supp}}{\Delta P\, A_{supp}} = \frac{1}{\frac{\mu}{\rho}\left(32\frac{L_{supp}}{D_{supp}^2} + \frac{24}{n\langle D^3\rangle}\right)} \tag{S20}$$

Plugging in values, we obtain a liquid water permeance of,

$$\frac{\dot{m}_{NG+supp}}{\Delta P\, A_{supp}} = 0.9\ \frac{\mathrm{g}}{\mathrm{m}^2\ \mathrm{day\ Pa}} \tag{S21}$$

In reasonable agreement with our measurements.

Supplementary Note 2. Molecular dynamics simulations – transport modeling

In this paper, we have employed a simple analytical transport model to explain the significantly different transport rates measured in water vapor and liquid water permeation experiments. This

modeling neglects many fascinating transport phenomena that have been uncovered in nanoflows. Here we more closely examine what impact such factors could have on the model predictions.

Supplementary Note 2.1. Molecular dynamics simulations of water vapor transport across nanoporous graphene membranes: methodology

Molecular dynamics simulations were performed to estimate the discrepancy between the simple Knudsen effusion model employed and the actual water vapor flow rates through sub-nanometer pores in graphene. These simulations capture such effects as adsorption of vapor molecules on graphene, surface diffusion of vapor molecules along the membrane, and the atomic scale geometry of the pore and water molecules during crossings.

Molecular dynamics simulations were performed in LAMMPS (Large-scale Atomic/Molecular Massively Parallel Simulator)[12] and visualized in VMD (Visual Molecular Dynamics).[13] A 44.2 Å × 59.5 Å graphene membrane spanned the width of the domain in two directions and was positioned in the center of the domain in the third direction (Supplementary Figure 13), the length of which was chosen to set the nominal pressure. Four identical, equally spaced holes were created in the graphene. The hole geometries simulated are shown in Supplementary Figure 14. Nine pores with carbon atoms on the pore rim were simulated (Supplementary Figure 14a-i, designated C-1 to C-9) matching those in Ref. [14], where similar permeance simulations were performed for other gas species. Four pores with hydrogen or hydroxyl groups on the pore rim were also simulated (Supplementary Figure 14j-m, designated F-1 to F-4) to increase the affinity of water vapor molecules to the pore rim and thereby capture the effects of high adsorption and test whether condensation could be induced within the pores. Periodic boundary conditions were imposed in all three directions. A second graphene sheet, this one without pores, was positioned parallel to the first at the end of the domain. This divides the domain into two equal volume reservoirs and prevents gas molecules from passing between the two except through the porous graphene membrane.

Fifty (50) water molecules were placed in the domain, half on one side of the porous graphene membrane and half on the other. A TIP4P water model[15] was used with a long-range Particle-Particle Particle-Mesh (pppm) Coulombic solver (masses [g/mol]: 15.9994 (oxygen), 1.008 (hydrogen); charges [e]: -1.0484 (oxygen), 0.5242 (hydrogen); O-H bond length [Å]: 0.9572; H-O-H bond angle [degrees]: 104.52; distance from oxygen to massless charge [Å]: 0.1250; O-O Lennard Jones epsilon [kcal/mol]: 0.16275; O-O Lennard Jones sigma [Å]: 3.16435; Lennard Jones cutoff radius [Å]: 12; O-H and H-H Lennard Jones sigma and epsilon values were set to zero). The SHAKE algorithm was used to hold the water molecules rigid during the simulations. Membrane molecules were fixed in position for the duration of the simulation.

The Lennard-Jones potential parameters and charge used to model interactions between atoms are listed in Supplementary Table 2. These parameters follow Ref. [16] based on Ref. [17] and [18]. Interaction parameters between dissimilar atoms were computed by Lorentz-Berthelot mixing rules. A cutoff radius of 12 Å was imposed on Lennard-Jones interactions.

Simulations were performed at 300 K, in the range measured in this study, and at 380 K, where higher vapor pressures can be simulated to increase the number of molecule crossings and reduce uncertainty in permeance. A time step of 1 fs was used. Initial water molecule velocities were

drawn from a Maxwell-Boltzmann distribution at the prescribed temperature. Simulations were performed in the NVT ensemble using a Nosé-Hoover thermostat. Replicate simulations for the same pore size were initiated using different random initial velocities. Supplementary Table 3 lists the number of replicate simulations and total simulation time for each pore and temperature.

The simulations were run at equilibrium, with both sides of the membrane at the same average pressure. Under these conditions, the water vapor is an ideal gas, so crossings in the forward and backward directions are approximately independent. The total number of crossings in both directions is recorded over time (e.g., Supplementary Figure 15). Molecule positions were recorded every 10,000 timesteps to calculate permeance. Crossings were counted each time a molecule passed from >10 Å away from the membrane on one side to >10 Å away from the membrane on the other side.

Permeation coefficient [molecules/s-Pa-pore] was calculated as,

$$\dot{N} = \frac{N}{2 \cdot 4 \cdot t \cdot P} \tag{S22}$$

where N is the number of crossings counted in both directions, P [Pa] is the gas pressure, t [s] is the simulated time, the factor of 2 accounts for crossings in both directions, and the factor of 4 accounts for there being four identical pores in the membrane.

The gas pressure in Eq. S22 was calculated from the ideal gas law. Gas adsorption to the graphene membrane and graphene separator effectively reduce the bulk pressure in the gas for this small system. For each pore size, the pressure in Eq. S22 was corrected for this by calculating the average number of molecules in the volumes more than 10 Å away from the membrane and separator. Averaging was performed over all replicate simulations for a given pore size. This volume, and the average number of molecules in this volume, were used in the ideal gas law to calculate the bulk pressure. This correction reduced the pressure by ~1%. The average pressure over all replicate simulations for each pore type is reported in Supplementary Table 3. In all simulations, the pressure corresponds to 55-56% relative humidity, based on saturation pressures of 3.6 kPa at 300 K and 129 kPa at 380 K.[19]

Flow rates are summarized in Supplementary Figure 16 and Supplementary Table 3. Pore areas (A) were calculated using a hit-and-miss Monte Carlo method to find the area not within $D_m/\sqrt{2}$ of any membrane atom, where D_m is the Lennard-Jones diameter of a membrane atom. Effective pore diameter was calculated as the diameter of a circle with the same area, *i.e.*, $D = \sqrt{4A/\pi}$. This is the method for calculating an effective graphene pore diameter proposed by Sun et al.[14] for gas transport, where the factor of $\sqrt{2}$ approximately accounts for gas molecule collisions with the pore rim that deflect the molecule into the pore.

Supplementary Note 2.2. Molecular dynamics simulations of water vapor transport across nanoporous graphene membranes: results

The molecular dynamics simulation results in Supplementary Figure 16 provide a way to estimate the error introduced by using a simple Knudsen effusion model for water vapor transport through

graphene nanopores. Supplementary Figure 16 presents pore permeation coefficient ($\dot{N}$ [molecules/s-Pa-pore]), which is related to the mass flow rate ($\dot{m}_{pore}$ [kg/s-pore]) by,

$$\dot{N} = \frac{\dot{m}_{pore}}{m\,\Delta P} \tag{S23}$$

In the case of Knudsen effusion, substituting Eq. S6 into S23 shows that,

$$\dot{N} = \frac{\pi}{4} D^2 \frac{1}{\sqrt{2\pi m k_B T}} \tag{S24}$$

This expression is plotted in Supplementary Figure 16 alongside the molecular dynamics results.

For the smallest carbon terminated pore simulated, steric hinderance reduces the permeance compared to the Knudsen effusion model by ~80%. For the other eight carbon terminated pores simulated, the Knudsen effusion model under-predicted permeance by up to ~70%. However, whereas the Knudsen effusion model assumes ballistic crossings of gas molecules from one side of the pore to the other, without collision, up to ~50% of the crossings from the molecular dynamics simulations result from an adsorption pathway, in which adsorbed vapor molecules diffuse along the membrane to the pore. This is shown by the “direct permeance” markers in Supplementary Figure 16, which present the contribution of ballistic crossings to the total permeance. This was calculated by counting the number of crossings in which a gas molecule passed from >10 Å from the membrane on one side to >10 Å from the membrane on the other side within 20 ps. We note that this approach is expected to under-count direct crossings because some slower moving gas molecules will take more than 20 ps to travel the 20 Å. From the Maxwell-Boltzmann distribution, this fraction of molecules is estimated to be 21% at 300 K and 19% at 380 K. The uncertainty introduced by this approach is included in the error bars presented in Supplementary Figure 16 for direct permeance. For all but the smallest carbon terminated pore simulated, the direct permeance computed from the simulations is within 30% of the Knudsen effusion model.

The UV/ozone generated graphene pores in this study are expected to have functional groups passivating the pore rim. These groups could increase the affinity of water vapor molecules for the surface, increasing adsorptive transport, or potentially causing condensation of water vapor in the pore. To investigate this possibility, we performed simulations on four graphene pores with hydrogen or hydroxyl terminal groups (pores F-1 to F-4) at 2000 Pa nominal pressure. The resulting flow rates are summarized in Supplementary Figure 16. Whereas the carbon terminated pores had flow rates less than 1.7 times higher than predicted by the Knudsen effusion model, flow rates through functionalized pores were up to 2.5 times higher. A smaller fraction of the permeance of the functionalized pores results from direct crossings (Supplementary Figure 16), indicating that increased adsorption on the membranes is contributing to this higher permeance.

Although the permeation rate by the adsorption pathway is enhanced for the membranes with functionalized pores, the greater affinity for these membranes did not cause condensation of water vapor within the pores. The average number of water molecules within a cylinder with the diameter of the pore and extending 10 Å above and below the pore was less than 0.03 for all pores simulated. Animations of all simulations were reviewed and at no point did groups of water molecules collect around the pores.

Supplementary Note 2.3. Summary of molecular dynamics liquid water permeation rates through graphene nanopores

To assess the error introduced by using a simple Sampson flow model for graphene nanopore liquid water permeance, we compiled the summary plot in Supplementary Figure 17 of various published molecular dynamics simulation results for liquid water permeation rates through graphene nanopores. This plot includes simulations of graphene pores with various terminal groups and using different water models, as identified in the caption. Although pore diameter definitions can vary, Supplementary Figure 17 presents the pore diameter stated in each source paper to avoid introducing errors while reconstructing pore geometries. This may introduce some data scatter.

The permeation coefficient ($\dot{N}$ [molecules/s-Pa-pore]) predicted by the Sampson model can be obtained by substituting $\dot{m}_{pore}$ [kg/s-pore] from Eq. S17 into Eq. S23, resulting in,

$$\dot{N} = \frac{D^3\,\rho}{24\,\mu\,m} \tag{S28}$$

This curve is plotted in Supplementary Figure 17 for comparison to the molecular dynamics data. In this calculation, values of $\rho = 1000$ kg/m^3 and $\mu = 0.001$ Pa-s were used.

To better align with molecular dynamics simulation data for liquid water permeance of graphene nanopores, Suk & Aluru[11] developed a fit to correct for factors such as slip, finite pore aspect ratio, and viscosity changes under nanoconfinement within the pore. In terms of permeation coefficient, their fit becomes,

$$\dot{N} = \frac{\pi\left[\left(\frac{D}{2}\right)^4 + 4\delta\left(\frac{D}{2}\right)^3\right]\rho}{8\,\mu\,L_h\,m} \tag{S29}$$

where,

$$L_h = 0.27\left(\frac{D}{2}\right) + 0.95\times10^{-9}\ \text{m} \tag{S30}$$

$$\delta = \frac{1.517\times10^{-19}\ \text{m}^2}{D/2} + 0.205\times10^{-9}\ \text{m} \tag{S31}$$

$$\mu = \frac{8.47\times10^{-13}\ \text{Pa s m}}{D/2} + 0.00085\ \text{Pa s} \tag{S32}$$

The difference in predicted permeation rate between this model and the Sampson model for a 10 Å pore diameter is 8%. The absolute error decreases for smaller pore diameters.

Factors such as slip and affinity for terminal groups on the pore rim can enhance the permeation rate compared to the Sampson model (Eq. S28). Fig. 3G shows up to ~3.2 times higher permeation rates compared to the Sampson model in some cases over the range 0 Å $< D <$ 10 Å, relevant here. Although, it should be noted that Prasad et al.[20] have also observed differences in liquid water permeation rates of nearly a factor of ~2 for the same pore due to different choices of water model used in the molecular dynamics simulation.

Supplementary Note 2.4. Model deficiencies

The simple Knudsen effusion model for vapor transport and Sampson flow model for liquid lose accuracy for pores similar in size to fluid molecules. They fail to capture several important transport mechanisms.[11,14,16,21–25] In liquid water, deviations from continuum transport occur at these pore sizes. Velocity slip, viscosity changes under nanoconfinement, and the formation of dense liquid layers near the membrane alter liquid flow rates. Similarly, the non-zero size of gas molecules and adsorption on the membrane alter vapor flow rates. The extent of these deviations are quantified by the molecular dynamics simulation results summarized in Supplementary Figure 15 and 16.

Fig. 3G provides a comparison of liquid water and water vapor flow rates through graphene nanopores from molecular dynamics simulations. Molecular dynamics simulations predict enhancements in both liquid water and water vapor flow rates in comparison to the Sampson and Knudsen effusion models by up to a factor of ~3. Although such differences are important in determining the precise permeance values, they do not account for the larger factor of ~80 difference between liquid and vapor transport rates. Fig. 3G illustrates that, for various graphene pore structures, water vapor permeance is orders of magnitude higher than liquid water permeance. This large difference results from the different flow regimes in which liquid water and water vapor transport occurs across the same graphene nanopore. Although the Knudsen effusion and Sampson flow models do not resolve the precise permeation rates, which are affected by the nanoscale details of the pore, they do capture this difference in flow regime. As such, they provide the correct order of magnitude of both liquid water and water vapor flow rates, and the approximate difference between the two.

In modeling the liquid water and water vapor permeation rate through the membranes in this study, the Knudsen effusion and Sampson flow models are employed. A difference in graphene permeance by a factor of ~3 resulting from the molecular scale details of the pore, would significantly change the predicted permeance. Most pores simulated show less deviation than this, but nevertheless, in modeling, this difference would be lost in the inaccuracy of estimating pore density. Of the 89 pores imaged by STEM in this study, only 16 were in the size range of that would be permeable to water and not plugged by POSS-polyamide. This limited sampling introduced uncertainty in both the pore size distribution and density. Enhancements in flow rates due to molecular scale details of the pore are of similar magnitude in liquid and vapor, so errors in pore density will affect the magnitude of liquid and vapor permeance without significantly changing the ratio between the two. In modeling, using a fitted pore density rather than the measured pore density could be an appropriate approach in light of this uncertainty. However, the measured pore density happened to provide quantitative agreement of the transport model to the measurements here (which provided a self-consistency check to the results and some validation for multi-experimental approach using the same membrane), so using a fitted pore density was not employed.

In addition to uncertainty in the pore size distribution and density, the exact pore structures, and their distribution, is unknown. This would be required for accurate molecular simulation of precise permeation rates. This creates challenges in more detailed modeling of the membranes measured in this study.

Despite these deficiencies, the Knudsen effusion and Sampson flow models serve the purpose of explaining the factor of ~80 difference between water vapor and liquid water flow rates as resulting primarily from the different flow regimes in which transport occurs. Although there are important aspects of transport that are uncertain, such as the terminal groups on the pore edge, and the precise interactions of the water molecules with the graphene, the modeling explains the measured trends quite well. While there are many fascinating transport phenomena that can occur through sub-nanometer pores in graphene that can affect transport rates, the magnitude of these effects is much smaller than the differences between liquid and vapor transport rates. Molecular dynamics simulations have found enhancements in flow rates due to nanoscale surface interactions by up to a factor of ~3. However, these differences fall far short of the factor of ~80 difference between liquid and vapor transport rates measured. This difference is well explained by the different flow regimes in which liquid and vapor transport occurs through graphene nanopores, and is captured by simple Knudsen effusion and Sampson flow models.

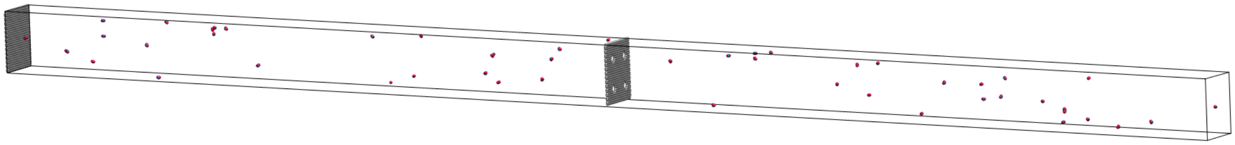

Supplementary Figure 13. Water vapor permeance molecular dynamics simulation snapshot illustrating domain setup. Water molecules (red hydrogen atoms and blue oxygen atoms) on both sides of a graphene (grey carbon) membrane containing four identical pores. Another graphene sheet without pores is positioned on the left end of the domain to separate the upstream and downstream reservoirs at that periodic boundary.

Supplementary Table 2. Potential parameters drawn from Ref. 16 **based on Ref.** 17 **and** 18**. Lennard-Jones parameters shown are between atoms of the same type. Interaction parameters between atoms of different types are calculated by Lorentz-Berthelot mixing rules.**

	**C (bonded only to carbon)**	**C_{COH}**	**H_{COH}**	**O_{COH}**	**C_{CH}**	**H_{CH}**	**O_{water}**	**H_{water}**
ε (kcal/mol)	0.0859	0.0703	0	0.155	0.046	0.0301	0.16275	0
σ (Å)	3.3997	3.55	0	3.07	2.985	2.42	3.16435	0
Charge, q (e)	0	0.2	0.44	-0.64	-0.115	0.115	-1.0484	0.5242

Supplementary Table 3. Summary of molecular dynamics simulation results for water vapor transport through graphene pores.

Pore designation	Effective pore diameter [Å]	Temperature [K]	Number of replicate simulations	Total simulated time [ns]	Total molecule crossings (sum in both directions)	Average pressure [kPa]	Permeation coefficient [molecule/ Pa-s-pore]
C-1	2.06	380	5	250	32	70.4	227
C-2	3.04	380	5	250	543	70.5	3851
C-3	3.78	380	5	250	845	70.5	5990
C-4	4.39	380	5	250	1065	70.6	7544
C-5	4.93	380	5	250	1213	70.7	8576
C-6	5.49	380	5	250	1460	70.5	10347
C-7	6.00	380	5	250	1622	70.7	11475
C-8	6.90	380	5	238	1939	70.6	14435
C-9	7.59	380	4	200	1888	70.4	16709
C-9	7.59	300	50	2500	974	1.99	24526
F-1	9.31	300	15	750	343	2.00	28637
F-2	7.67	300	15	750	319	1.99	26653
F-3	4.78	300	15	750	65	1.99	5430
F-4	3.45	300	15	750	98	1.99	8194

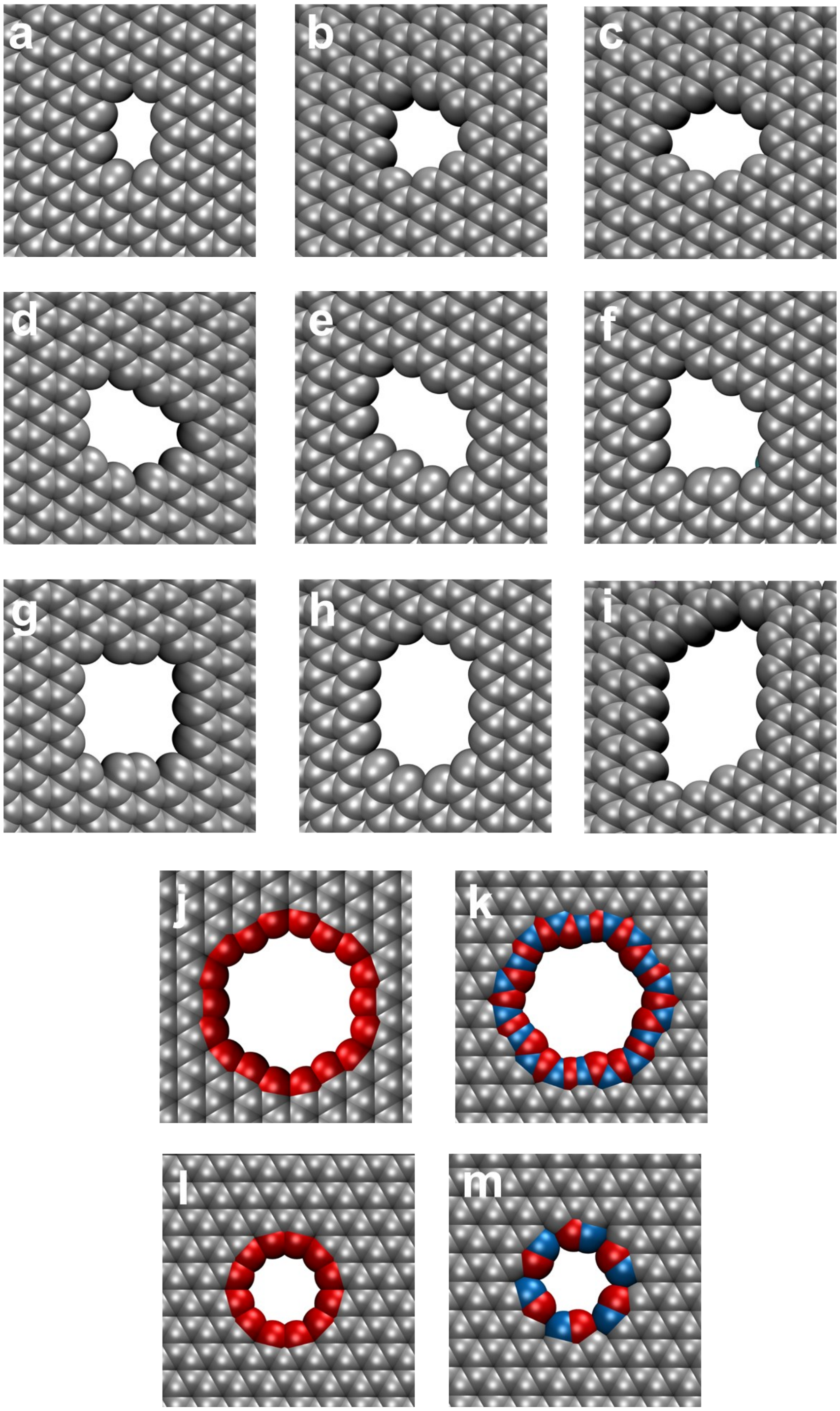

Supplementary Figure 14. Pore geometries used in molecular dynamics simulations. Designations: **a** C-1, **b** C-2, **c** C-3, **d** C-4, **e** C-5, **f** C-6, **g** C-7, **h** C-8, **i** C-9, j F-1, **k** F-2, **l** F-3, **m** F-4. Grey is carbon, red is hydrogen, blue is oxygen.

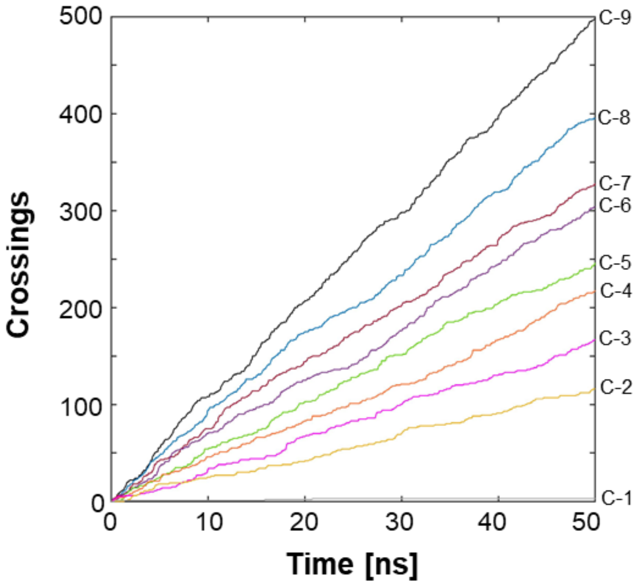

Supplementary Figure 15. Example time traces of total number of molecule crossings (sum of both directions). Each trace corresponds to a simulation for a different pore at 380 K, with the pore designation indicated on the right.

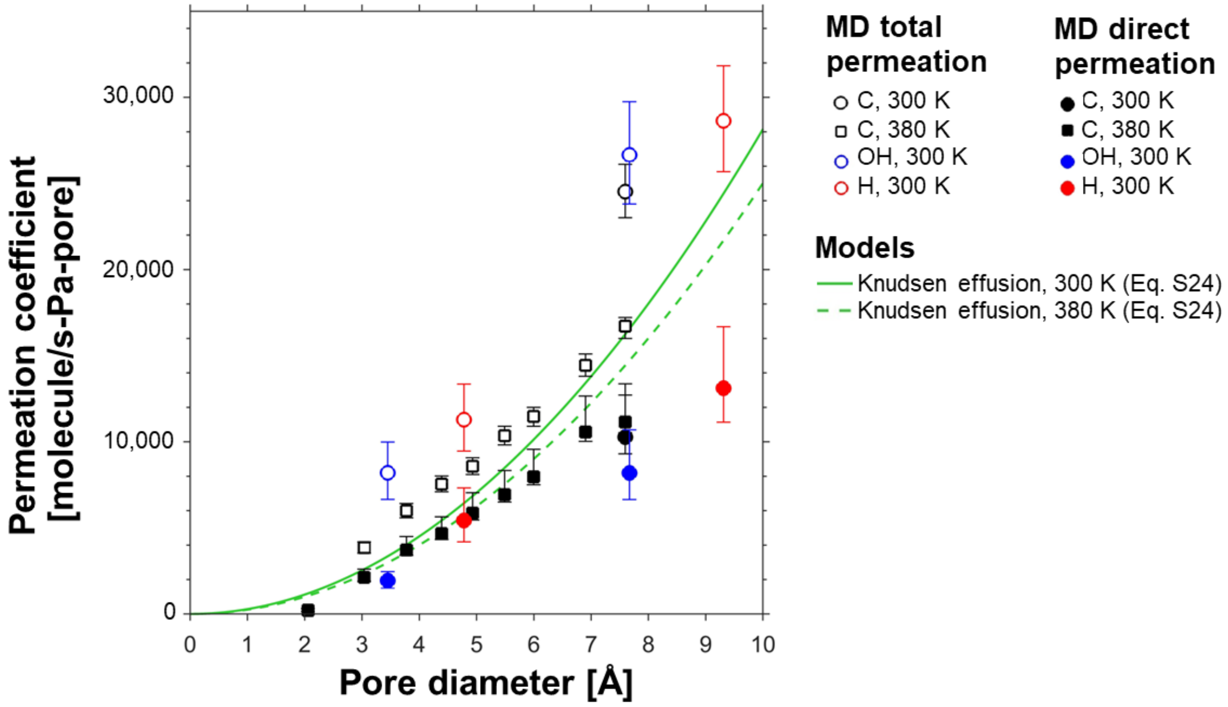

Supplementary Figure 16. Water vapor molecular permeation coefficients from molecular dynamics simulations. The total permeation coefficient (unfilled markers), which includes all molecule crossings, is plotted along with direct permeation coefficient (filled markers), which

counts only those molecules that passed from >10 Å away on one side of the membrane to >10 Å away on the other side of the membrane in less than 20 ps. Uncertainty bars for total permeance show the 95% confidence interval for a Poisson process. Uncertainty bars for direct permeance account for both the 95% confidence interval for a Poisson process and for an up to 21% (19%) under-estimation of the direct crossings at 300 K (380 K) due to the fraction of molecules moving slowly enough that 20 ps would not be sufficient time to directly cross a 20 Å gap. Simulations were performed at a relative humidity of 55 to 56%. Legend format for markers is "pore terminal groups, temperature."

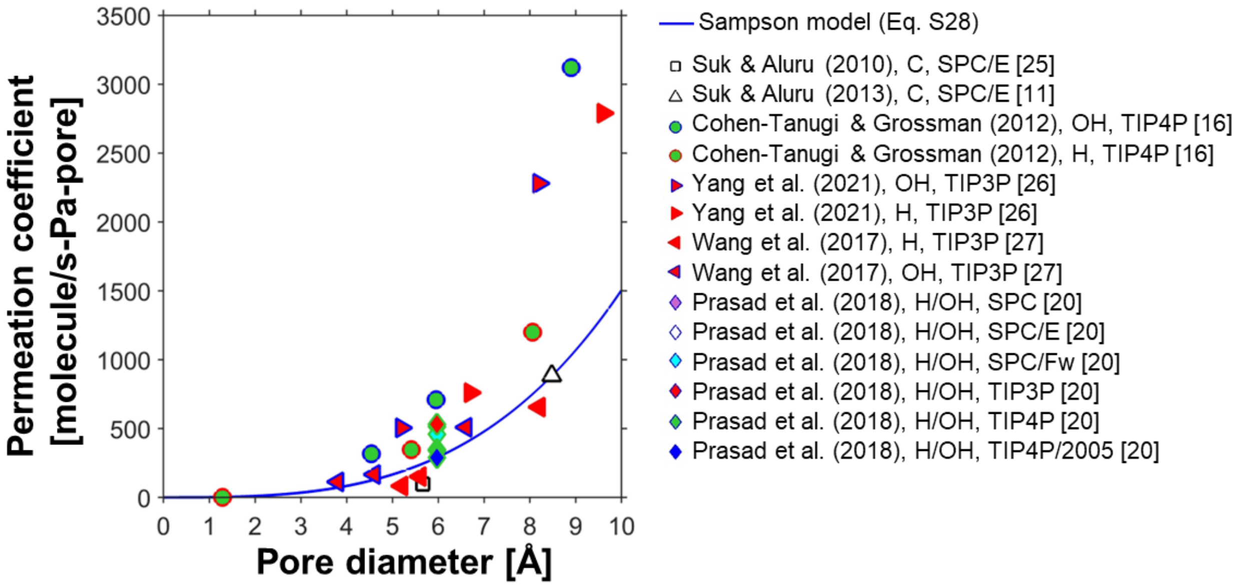

Supplementary Figure 17. Compilation of published molecular dynamics simulation predictions for water permeation through graphene nanopores.[11,16,20,25–27] The pore diameter plotted is that stated in the corresponding paper. Legend format for markers is "authors (year), pore terminal groups, water model [citation number]." Also see Fig. 3G.

Supplementary References

1. Bui, N. *et al.* Ultrabreathable and Protective Membranes with Sub-5 nm Carbon Nanotube Pores. *Adv. Mater.* **28**, 5871–5877 (2016).
2. Yang, Y. *et al.* Large-area graphene-nanomesh/carbon-nanotube hybrid membranes for ionic and molecular nanofiltration. *Science* **364**, 1057–1062 (2019).
3. Wang, L. *et al.* Fundamental transport mechanisms, fabrication and potential applications of nanoporous atomically thin membranes. *Nat. Nanotechnol.* **12**, 509–522 (2017).
4. Jang, D., Idrobo, J.-C., Laoui, T. & Karnik, R. Water and Solute Transport Governed by Tunable Pore Size Distributions in Nanoporous Graphene Membranes. *ACS Nano* **11**, 10042–10052 (2017).
5. O'Hern, S. C. *et al.* Nanofiltration across Defect-Sealed Nanoporous Monolayer Graphene. *Nano Lett.* **15**, 3254–3260 (2015).
6. Cheng, P. *et al.* Facile Size-Selective Defect Sealing in Large-Area Atomically Thin Graphene Membranes for Sub-Nanometer Scale Separations. *Nano Lett.* **20**, 5951–5959 (2020).
7. Bird, R. B., Stewart, W. E. & Lightfoot, E. N. *Transport phenomena*. (2006).
8. Bird, G. A. *Molecular gas dynamics and the direct simulation of gas flows*. (1994).
9. White, F. M. *Viscous fluid flow*. (McGraw-Hill Higher Education, 2006).
10. Sampson, R. A. & Greenhill, A. G. On Stokes's current function. *Philos. Trans. R. Soc. London.* **182**, 449–518 (1891).
11. Suk, M. E. & Aluru, N. R. Molecular and continuum hydrodynamics in graphene nanopores. *RSC Adv.* **3**, 9365–9372 (2013).
12. Plimpton, S. Fast Parallel Algorithms for Short-Range Molecular Dynamics. *J. Comput. Phys.* **117**, 1–19 (1995).
13. Humphrey, W., Dalke, A. & Schulten, K. VMD: Visual molecular dynamics. *J. Mol. Graph.* **14**, 33–38 (1996).
14. Sun, C. *et al.* Mechanisms of Molecular Permeation through Nanoporous Graphene Membranes. *Langmuir* **30**, 675–682 (2014).
15. Abascal, J. L. F. & Vega, C. A general purpose model for the condensed phases of water: TIP4P/2005. *J. Chem. Phys.* **123**, 234505 (2005).
16. Cohen-Tanugi, D. & Grossman, J. C. Water Desalination across Nanoporous Graphene. *Nano Lett.* **12**, 3602–3608 (2012).
17. Mooney, D. A., Müller-Plathe, F. & Kremer, K. Simulation studies for liquid phenol: properties evaluated and tested over a range of temperatures. *Chem. Phys. Lett.* **294**, 135–142 (1998).
18. Müller-Plathe, F. Local Structure and Dynamics in Solvent-Swollen Polymers. *Macromolecules* **29**, 4782–4791 (1996).
19. Cengel, Y. A. & Boles, M. A. *Thermodynamics : an engineering approach*. (McGraw-Hill, 2006).
20. K., V. P., Kannam, S. K., Hartkamp, R. & Sathian, S. P. Water desalination using graphene nanopores: influence of the water models used in simulations. *Phys. Chem. Chem. Phys.* **20**, 16005–16011 (2018).
21. Faucher, S. *et al.* Critical Knowledge Gaps in Mass Transport through Single-Digit Nanopores: A Review and Perspective. *J. Phys. Chem. C* **123**, 21309–21326 (2019).
22. Kavokine, N., Netz, R. R. & Bocquet, L. Fluids at the Nanoscale: From Continuum to Subcontinuum Transport. *Annu. Rev. Fluid Mech.* **53**, 377–410 (2021).

23. Drahushuk, L. W. & Strano, M. S. Mechanisms of Gas Permeation through Single Layer Graphene Membranes. *Langmuir* **28**, 16671–16678 (2012).
24. Wong, A. O. K., Atwal, H. K. & Boutilier, M. S. H. Molecular advection–diffusion through graphene nanopores. *Eur. J. Mech. - B/Fluids* **94**, 366–374 (2022).
25. Suk, M. E. & Aluru, N. R. Water Transport through Ultrathin Graphene. *J. Phys. Chem. Lett.* **1**, 1590–1594 (2010).
26. Yang, J., Shen, Z., He, J. & Li, Y. Efficient separation of small organic contaminants in water using functionalized nanoporous graphene membranes: Insights from molecular dynamics simulations. *J. Memb. Sci.* **630**, 119331 (2021).
27. Wang, Y., He, Z., Gupta, K. M., Shi, Q. & Lu, R. Molecular dynamics study on water desalination through functionalized nanoporous graphene. *Carbon* **116**, 120–127 (2017).
